# The Impact of COVID-19 on Physical Activity and Life Satisfaction of Golfers: A Cross-Sectional Study in German-Speaking Countries

**DOI:** 10.3389/fspor.2022.913244

**Published:** 2022-07-12

**Authors:** David Jungwirth, Martin Angerer, Daniela Haluza

**Affiliations:** Department of Environmental Health, Center for Public Health, Medical University of Vienna, Vienna, Austria

**Keywords:** COVID-19 pandemic, physical activity, health, nature, sitting time, exercise

## Abstract

Golf is an outdoor sport played worldwide, and golfers of all ages benefit from regular exercise and social contact. The COVID-19 crisis has led to lockdowns, curfews, and closures of golf courses and other indoor and outdoor sports facilities. This study aimed to retrospectively assess how golfers perceived the impact of the crisis on physical activity and life satisfaction. We conducted a cross-sectional online survey among 1,115 golfers (61% men, mean age 55.0 years) in German-speaking countries, mainly Austria and Germany, from March to June 2021. When comparing ratings before and during the COVID-19 period, participants reported exercising more indoors at home independently or with online instructions. Likewise, the popularity of exercising outdoors in publicly accessible open spaces increased overall, especially in rural areas by about 10%. Life satisfaction decreased significantly by 24.2% (*p* < 0.001) during the pandemic. From a public health perspective, access to outdoor sports facilities such as golf courses might alleviate the negative psychosocial and health effects of home confinement and restricted access to indoor sports facilities. Further research is recommended to evaluate the longer-term impact of COVID-19-related measures on the physical activity and life satisfaction of golfers.

## Introduction

Regular physical activity is essential for promoting health and preventing noncommunicable diseases (Fiuza-Luces et al., 2018). Thus, replacing sedentary time with physical activity of any intensity (including light intensity) provides health benefits. The WHO 2020 guidelines on physical activity and sedentary behavior, targeting adults 18–64 years old, recommend at least 150–300 min per week of moderate- to high-intensity physical activity for sustainable benefits for mental health, immune system function, and longevity (Bull et al., 2020). Evidence is accumulating that natural environments provide motivation to bephysically active (Haluza et al., 2014; Cervinka et al., 2020). In addition, spending time in nature helps people to recover from stress by reducing the allostatic load, thus improving human health and wellbeing (Haluza et al., 2014). A systematic review by Manferdelli and colleagues found that outdoor activities also prevent vitamin D deficiency, osteoporosis, and myopia (Manferdelli et al., 2019). In addition, outdoor sports are linked to societal benefits for individuals and communities as a whole, as public sports venues like parks and other green spaces are easily accessible (Eigenschenk et al., 2019).

The broad range of positive social, psychological, and physiological effects mediated by the restorative effects of exposure to outdoor nature cannot be seen with similar physical activities practiced indoors (Brito et al., 2022). Golf, a sport predominantly played outdoors, is likely to advance quality of life, stress management, and health perception (Eigenschenk et al., 2019). Due to improved cardiovascular, respiratory, and metabolic health, bone density, balance, fall prevention, and mood and mental wellbeing, the sport of golf contributes to increased satisfaction with life (Sorbie G. et al., 2020; Sorbie G. G. et al., 2020). Life satisfaction is the subjective evaluation of individuals regarding the extent to which their life as a whole—in other words, whether their needs, goals, and wishes are met.

A set of aspects viewed as unique to golf include that it is an organized sport with opportunities for social and community engagement, time for self, but also time spent with others (Stenner et al., 2016). In addition, the relatively well-adjustable intensity of the physical effort allows golfers to play irrespective of age and physical abilities, provoking the impression to exercise without feeling it like exercise. Golf-related activities correspond to a mainly low to moderate physical activity, depending on personal fitness and golf course-related requirements (Luscombe et al., 2017). Notably, golfers who use electronic golf carts are still physically active, hence walking distances covered and physical training effects during a golf session are lower compared to unaided walking (Murray et al., 2017). However, the possibility to use golf carts could be a valuable argument for people with disabilities or restricted walking abilities. For these reasons, golf is a popular sport among people over 55 years (Stenner et al., 2016). Another unique feature of golf makes it an exceptionally fair and inclusive sport: the handicap system. A golf handicap is a numerical measure of a golfer's potential used to enable players of varying abilities to compete equally, with better players having lower handicaps (Golf Handicap Guide, 2014).

In March 2020, the COVID-19 pandemic caused massive restrictions on everyday social life as we knew it (Riou and Althaus, 2020). Due to stay-at-home orders, lockdowns, and social distancing measures, indoor and outdoor sports facilities like fitness centers, sports clubs, and golf courses were closed for many weeks or even months (Wilder-Smith and Freedman, 2020; Mutz and Gerke, 2021). Recently, many international studies reported on the negative impact of COVID-19 on the quantity of physical activity, while screen-related sedentary behavior increased (Gallo et al., 2020; Pieh et al., 2020; Katewongsa et al., 2021). There is also scientific evidence that quality of life, mental health, and wellbeing decreased due to the COVID-19 pandemic in all strata of the population worldwide (Pieh et al., 2020; Duan et al., 2021). Mirroring the risk of psychosocial strain during lockdowns, decreased life satisfaction scores were commonly found in studies on COVID-19 effects (Ammar et al., 2020b; Zhang et al., 2020; Eek et al., 2021; Jungwirth et al., 2021). Potential reasons include decreased social contacts with family and friends, restrictions in mobility, and closed sports facilities (Zhou and Lin, 2016; Ammar et al., 2020a,b; Haider et al., 2021; Stieger et al., 2021). Exemplarily, a Swedish study from autumn 2020 reported that decreased levels of physical activity also decreased life satisfaction (Eek et al., 2021). As proposed by Wheatley and Bickerton, playing golf can yield higher levels of life satisfaction compared to other sports and leisure activities normally performed at moderate intensity (Wheatley and Bickerton, 2017). Sorbie G. et al. (2020) showed that golfers had significantly higher life satisfaction levels compared to the English population. In this line, life satisfaction significantly improved when golfing restrictions were relaxed after the first lockdown in the United Kingdom (Sorbie et al., 2021).

In line with previous studies on the health and wellbeing benefits of exposure to outdoor nature, people were eventually allowed to exercise outdoors during COVID-19 restrictions (Haluza et al., 2014; Kondo et al., 2018; Cervinka et al., 2020; Stieger et al., 2021). Empirical evidence on the importance of public urban green spaces and periurban nature as a cost-free, easy admissible alternative for indoor and outdoor sports facilities closed due to the crisis was collected (Haider et al., 2021; Jungwirth et al., 2021; Reinwald et al., 2021; Stieger et al., 2021). However, golf is a club-and-ball sport played on a widespread course with an arranged progression of normally 18 holes, and golfers aim at hitting balls into a series of holes on this course in as few strokes as possible. Therefore, the specific features of the sport encompassing a wide area of manicured lawn with defined holes are unlikely to find adequate replacements in public spaces.

Acknowledging the positive effects of outdoor sports on the example of golf, this study, conducted in 2021, i.e., the second year of the COVID-19 crisis, adds so far lacking knowledge on behavioral and perceptive changes to the rapidly increasing amount of scientific reports on physiopsychosocial effects of the pandemic (Zhou and Lin, 2016; Haider et al., 2021; Robinson et al., 2021a; Stieger et al., 2021). The present cross-sectional online study surveyed a nonrepresentative convenience sample of German-speaking golfers. By comparing ratings before vs. during the pandemic, we aimed at elucidating whether the COVID-19 pandemic (i) led to a shift in using sports facilities for physical activity from indoor to outdoor spaces, (ii) changed reasons for being outdoors, and (iii) affected life satisfaction.

## Methods

### Study Design

The present non-representative, cross-sectional online study assessed self-reported prevailing perceptions regarding physical activity and life satisfaction in the context of the COVID-19 crisis among German-speaking adult golfers. So, the inclusion criteria were (Fiuza-Luces et al., 2018) age 18 or over (Bull et al., 2020), playing golf (Haluza et al., 2014), German language proficiency, and (Cervinka et al., 2020) ability to fill out an online survey. The survey was designed using the Checklist for Reporting Results of Internet E-Surveys (Eysenbach, 2004). Participation was voluntary, and we did not offer incentives. We did not use randomized or adaptive items. Before data collection, full ethical approval was granted from the institutional ethical committee of the Medical University of Vienna, Austria, on 1 April 2021. The study was conducted following the ethical standards laid down in the Declaration of Helsinki.

To review completeness and comprehensibility, 15 voluntary participants, golfers and nongolfers, pretested the online survey in March 2021. The adapted online survey was accessible barrier-free *via* the web-based survey tool SoSci Survey from 1 April to 6 June 2021^1^. The cover page informed participants about the study's aim, and they gave written informed consent before starting the survey. An explanatory text informed participants that, for this survey, the period “before COVID-19” comprised the time before 16 March 2020, and “during COVID-19” the time after this date (Pieh et al., 2020). The online survey link was spread *via* the commonly used social networks Facebook and WhatsApp of several golf organizations, and golf clubs, such as Perfect Eagle Golf^2^, the Austrian Golf Association^3^, and the German Golf Association^4^.

### Measures

The first part of the online survey collected data on sociodemographic characteristics age (in years), gender (male, female, and divers), country of residence (Austria, Germany, Switzerland, other neighboring German-speaking countries, i.e., Lichtenstein or Luxembourg, and others), and education level (primary, secondary, and tertiary). We further asked for a golf experience, in years (0–5, 6–10, and 11 years or longer), and a golf handicap index (Club standard/Category 6: 54–37, Category 5: 36–26.5, Category 4: 26.4–18.5, Category 3: 18.4–11.5, Category 2: 11.4–4.5, and Category 1: 4.4 or less, I do not know), according to the European Golf Association handicap system (Golf Handicap Guide, 2014).

We further assessed ratings on the frequency of physical activity in three different indoor facilities (fitness center, at home independently, and also at home with online instructions) and five outdoor (urban area, public green space, periurban forest, rural area, and golf course) places using items of the Eurobarometer special survey 334^5^. Each of these activities was separately assessed before and during the COVID-19 pandemic using a 5-point Likert scale ranging from 1 to 6: 1: never, 2: ≤ 1 h per week, 3: 1 h per week, 4: 1–2 h per week, 5: 3–5 h per week, and 6: ≥5 h per week.

Participants rated six reasons for being outdoors (urge to move, recreation, social contacts, physical health, mental health, and distraction) before and during the COVID-19 pandemic on a 5-point Likert scale (1: strongly disagree, 2: disagree, 3: neutral, 4: agree, and 5: strongly agree) (Jungwirth et al., 2021).

The last part of the questionnaire assessed participants' life satisfaction before and during the COVID-19 pandemic using the standardized Short Life Satisfaction Questionnaire for Lockdowns (SLSQL) (Ammar et al., 2020a,b; Jungwirth et al., 2021). The SLSQL is a concise and convenient tool based on these three items: “In most ways my life is close to my ideal,” “So far, I have gotten the important things I want in life,” and “I am satisfied with my life,” with the response options ranging from 1 to 5 (1: strongly disagree, 2: disagree, 3: neutral, 4: agree, and 5: strongly agree).

### Statistical Data Analysis

A cross-sectional study design with a purposive sampling approach was used in this study. We calculated the sample size using G^*^Power 3.1.9.7 software for paired samples *T*-tests (Faul et al., 2007). For achieving a small effect size (Cohen's *d* = 0.15) for life satisfaction change as in previous studies (Ammar et al., 2020a,b; Jungwirth et al., 2021), with a two-sided alpha of 0.05 and a statistical power of 95%, a total of 580 participants was required.

We used descriptive statistics to report categorical data as absolute and relative frequencies and continuous data as mean and SD. We performed all statistical analyses using the statistical software SPSS Statistics for Windows, Version 27.0 (Armonk, NY, USA, IBM Corp.). We set statistical significance at *p* < 0.05. We employed paired samples *T*-tests to calculate changes in responses comparing the before and the during COVID-19 period. We calculated the effect sizes using Cohen's *d* to define the magnitude of the change score and interpreted them as small (i.e., 0.2), moderate (i.e., 0.5), or large (i.e., 0.8).

## Results

### Study Population

The web link to the online survey was accessed 3,269 times; 1,373 participants started and 1,123 of those fully completed the survey (81.79% completion rate). We excluded eight data sets from people who indicated to do not play golf, as we were interested in views of golfers only. Thus, the final sample included 1,115 participants, with a higher proportion of men (*n* = 680, 61.0%) compared to women (*n* = 435, 39.0%, Table 1). No participant indicated to be of diverse gender. The average age of study participants was mean 54.58 years (SD 13.37, range 18–85 years). The average completion time of the survey was a mean of 8.16 min SD 3.34. Most study subjects lived in Austria (64.9%), the rest in Germany (33.0%), and only very few participants lived in other countries. Furthermore, 24.5% (*n* = 273) of participants reported having a primary, 37.5% (*n* = 418) a secondary, and 34.3% (*n* = 383) a tertiary education level. As for golf experience, the majority of participants (83%) played golf for at least 6 years and had a handicap index of category 3 or better (76.1%).

**Table 1:** Sociodemographic characteristics of the study population (*n* = 1,115).

	***N***	**%**
Gender		
Female	435	39.0
Male	680	61.0
**Education level**		
Primary education	273	24.5
Secondary education	418	37.5
Tertiary education	383	34.3
Other	41	3.7
**Country of residence**		
Austria	724	64.9
Germany	368	33.0
Other	23	2.1
Golf experience		
0–5 years	190	17.0
6–10 years	265	23.8
11 years or longer	660	59.2
Handicap index*		
Club standard/ Category 6: 54–37	97	8.7
Category 5: 36–26.5	167	15.0
Category 4: 26.4–18.5	343	30.8
Category 3: 18.4–11.5	257	23.0
Category 2: 11.4 to 4.5	174	15.6
Category 1: 4.4 or less	75	6.7
I don't know	2	0.2
Total	1,115	100.0

*European Golf Association Handicap System.

### Frequency of Physical Activity

Table 2 reports the frequency of physical activity at specific indoor and outdoor sports facilities, each before and during the COVID-19 pandemic. We revealed statistically significant changes for all facilities (all *p* < 0.05). The frequency of fitness center use decreased by 42.12%, with a moderate effect size (*t* = 22.578, *d* = 0.676, *p* < 0.001). Before the crisis, 23.0% of participants (= 257) played golf for 3–5 h per week, and 64.0% (*n* = 714) for more than 5 h per week. Due to the reduced access to golf courses, the frequency of golf course use decreased by 7.77%, with a small effect size (*t* = 10.263, *d* = 0.307, *p* < 0.001). In contrast, we found a statistically significant average increase of physical activity indoors at home independently and with online instructions (14.18 and 23.48%, respectively, both *p* < 0.001), with moderate effect sizes. Outdoor activities in urban areas, public green spaces, periurban forests, and rural areas also increased statistically significantly, hence with smaller increases ranging from 3.16 to 10.39%.

**Table 2:** Frequency of physical activity at specific outdoor sports facilities before and during the COVID-19 pandemic among golfers (*n* = 1,115).

**Items**	**Before COVID-19**		**During COVID-19**					
	**Mean**	**SD**	**Mean**	**SD**	**Diff. %**	***T*-test**	**Cohen's *d***	***p*-value**
**Indoor sports facilities**								
Fitness center	1.92	1.23	1.11	0.49	−42.12	22.578	0.676	<0.001**
Indoor at home independently	2.15	1.13	2.51	1.25	14.18	13.534	0.405	<0.001**
Indoor at home with online instructions	1.31	0.72	1.71	1.08	23.48	15.359	0.460	<0.001**
**Outdoor sports facilities**								
Urban area	1.23	0.68	1.27	0.76	3.29	2.132	0.064	0.033*
Public green space	1.28	0.75	1.32	0.83	3.16	2.042	0.061	0.041*
Periurban forest	1.48	0.93	1.54	1.01	4.31	3.030	0.091	0.002*
Rural area	2.30	1.29	2.53	1.43	10.39	8.096	0.242	<0.001**
Golf course	4.44	0.91	4.10	1.18	−7.77	10.263	0.307	<0.001**

Means and SD; frequency of physical activity: 1: never, 2: ≤ 1 h per week, 3: 1 h per week, 4: 1–2 h per week, 5: 3–5 h per week, and 6: ≥5 h per week; p values from paired samples t test, *p < 0.05, **p < 0.001.

### Reasons for Being Outdoors

Table 3 depicts ratings on reasons for being outdoors before and during the COVID-19 crisis. We found a small, but statistically significant increase for all reasons for being outdoors except for “social contacts” (4.34% decrease), with all *p*-values smaller than 0.001 from paired samples *T*-tests for these changes. The most pronounced change before vs. during the COVID-19 pandemic was identified as the “urge to move,” with a small effect size (6.11%, *t* = 12.511, *d* = 0.375). For other reasons, the effect sizes of the changes were negligible.

**Table 3:** Reasons for being outdoors before and during the COVID-19 pandemic (*n* = 1,115).

**Items**	**Before COVID-19**		**During COVID-19**					
	**Mean**	**SD**	**Mean**	**SD**	**Diff. %**	***T*-test**	**Cohen's *d***	***p*-value**
Urge to move	4.15	0.91	4.41	0.82	6.11	12.511	0.375	<0.001**
Recreation	4.23	0.80	4.30	0.83	1.72	3.890	0.117	<0.001**
Social contacts	3.57	1.04	3.42	1.26	−4.34	4.259	0.128	<0.001**
Physical health	4.21	0.83	4.32	0.82	2.58	5.234	0.157	<0.001**
Mental health	4.20	0.83	4.29	0.82	2.35	4.902	0.147	<0.001**
Distraction	3.96	0.90	4.12	0.97	3.94	5.942	0.178	<0.001**

Means and SD; agreement: 1: strongly disagree, 2: disagree, 3: neutral, 4: agree, 5: strongly agree; **p-values from paired samples T-tests: < 0.001.

### Life Satisfaction in Golfers

Table 4 summarizes the responses to the SLSQL, comparing ratings before and during COVID-19. Internal consistency (Cronbach's alpha) for the three SLSQL items before COVID-19 was moderate (i.e., 0.799) and for the three SLSQL items during COVID-19 was high (i.e., 0.857). Paired samples *T*-tests showed significant decreases for all items, with all *p-*values smaller than 0.001. The total score of SLSQL, i.e., life satisfaction, decreased statistically significantly by 24.21% with a high effect size during compared to before COVID-19 (*t* = 35.515, *p* < 0.001, *d* = 1.064).

**Table 4:** Responses to the Short Life Satisfaction Questionnaire-Lockdowns (SLSQL) before and during the COVID-19 pandemic (*n* = 1,115).

**Items**	**Before COVID-19**		**During COVID-19**					
	**Mean**	**SD**	**Mean**	**SD**	**Diff. %**	***T*-test**	**Cohen's *d***	***p*-value**
In most ways my life is close to my ideal.	4.14	0.69	2.86	1.07	−30.92	37.502	1.123	<0.001**
So far, I have gotten the important things I want in life.	4.13	0.73	3.35	1.10	−18.89	25.466	0.763	<0.001**
I am satisfied with my life.	4.37	0.66	3.36	1.18	−23.11	29.643	0.888	<0.001**
Total score life satisfaction	4.21	0.59	3.19	0.99	−24.21	35.515	1.064	<0.001**

CI, confidence interval; agreement with the items: 1: strongly disagree, 2: disagree, 3: neutral, 4: agree, 5: strongly agree; ** all p-values from paired samples T-tests: < 0.001.

## Discussion

To the best of our knowledge, this is the first online cross-sectional study examining the effects of the COVID-19 pandemic on the use of indoor to outdoor spaces for physical activities, reasons for being outdoors, and life satisfaction in a large sample of German-speaking golfers. We compared ratings from before to those during the crisis. The findings from the study have fully supported the assumptions that the closures of golf courses led to a statistically significantly increased use of alternative modes of recreational activities indoors at home or outdoors in public spaces to fulfill the urge to move during crisis-related restrictions. Life satisfaction decreased in participants.

As golf is played by millions of people worldwide, the impact of COVID-19-related golf course closures on the health and wellbeing of golf enthusiasts is of high interest to individuals, sports organizations, golf clubs, public health experts, and decision-makers. The sport of golf as such has unique characteristics. First, golfers of all fitness levels profit from a vast range of physical health and mental wellbeing benefits mediated by constant moderate-intensity activity while walking and putting. Second, golf is played outdoors and is highly dependent on weather conditions and terrains. Notably, contact with natural environments has been linked to health and wellbeing (Haluza et al., 2014). Third, golfers often play with acquaintances, thereby creating and cultivating social relationships, and potentially also business networks. Last but not least, the handicap system allows for exceptional fair competition between two players of any level (Golf Handicap Guide, 2014).

With the closure of recreational facilities and fitness centers, outdoor or home-based exercise is of utmost importance. We assumed that reduced access to all sports facilities would motivate people to exercise outdoors and visit natural spaces more often for recreational purposes (Cervinka et al., 2020). Indeed, in our study, the frequency of outdoor activities in urban areas, public green spaces, periurban forests, and rural areas increased. We found changes in reasons for being outdoors when comparing before to during COVID-19 ratings, with the urge to move showing the strongest increase of about 6%, although with a small effect size. Furthermore, physical activity indoors at home independently and with online instructions increased significantly during the crisis. Home-based exercise was already popular before the pandemic, and its positive impact on physical and mental health has been examined and proven in various settings (Dwyer et al., 2020). Especially the online fitness sector experienced a boom in the last couple of years, and lockdowns and closures of sports facilities reinforce this global trend, as also seen in the surveyed golfers (Jungwirth et al., 2021).

As seen in other popular sports such as tennis, basketball, baseball, and soccer, watching golf tournaments live or on screen is also a common practice among golfers. Sedentary golf activities were also popular during the pandemic, with more than 70% of golfers in the United Kingdom watching golf on TV and as much as 84% performing golf-related activities at home, such as practicing golf swings, and putting (Sorbie G. G. et al., 2020). These physical activities form a crucial part of the game of golf. However, they do not include elements of walking, social engagement, exposure to outdoor nature, and direct competition with others, and thus fail to fully mirror the sport of golf and its enjoyment and pleasure. Although golf courses reopened periodically between the lockdowns, strict restrictions, namely, mandatory use of face masks in distinct places, social distancing rules, and restricted locker room and clubhouse access very likely reduced the social interactions that are important for many golfers (Robinson et al., 2021a).

In our study, golfers were quite experienced and skilled in terms of years of playing and handicap index. They also were not much interested in sports other than golf. Before COVID-19, more than 60% of participants played golf for more than 5 h per week, whereas other sports facilities such as indoor fitness centers were much less frequently used. Interestingly, the self-reported frequency of using golf courses did not dramatically decrease during the crisis, only by about 8% and showing a small effect size. Interpretations could be that regionally and for distinct periods of time golf courses were re-opened, especially in the spring and summer months of the years 2020 and 2021 (Sorbie et al., 2021).

In this study, we found a situational reduction in life satisfaction, with −24% and a large effect size in the total score of life satisfaction in the pre-during comparison. Notably, scores for the item “In most ways my life is close to my ideal.” decreased by more than 30%, showing a large effect size. This reduction is quite high in direct comparison with other studies using the same survey tool. An international survey launched in April 2020, found relative reductions of 18% for this item and 16% for the total score of life satisfaction (Ammar et al., 2020b), and an Austrian survey launched in March 2021, found decreases of −26% for this item's score and −20% for the total score life satisfaction (Jungwirth et al., 2021). Physical inactivity due to lockdowns, self-isolation, quarantine, homeschooling, and homeworking not only impacted fitness levels; but decreased scores in life satisfaction are commonly found in studies internationally (Ammar et al., 2020a,b; Zhang et al., 2020; Eek et al., 2021; Jungwirth et al., 2021). Zhang and co-workers found life satisfaction decreases and distress increases in working people in China due to the exceptional disruptions of lives and work caused by COVID-19 (Zhang et al., 2020).

With significant negative effects of the current COVID-19 pandemic on social participation and life satisfaction scores, the findings of our study support these previous reports, elucidating the risk of psychosocial strain during the COVID-19 period. Golf increases life satisfaction to a higher extent than other sports and leisure activities (Wheatley and Bickerton, 2017). Notably, Sorbie G. et al. (2020) showed in two studies that golfers have significantly higher life satisfaction levels compared to the general English population, and that the reopening of golf courses increased the life satisfaction of golfers (Sorbie et al., 2021). We found a drop in life satisfaction of about a fourth when comparing levels of the before to the during COVID-19 periods in our study participants.

Sports practitioners of all levels should follow an exercise regime close to their abilities and previous habits (Dwyer et al., 2020). International fitness guidelines suggest more than 300 min of moderate-intensity or more than 150 min of vigorous-intensity aerobic physical exercise per week, dependent on age, and other individual demographic characteristics (Warburton and Bredin, 2017).

Golf is usually played regularly and for a longer time, with several hours at a time being no exception. Taken together, golf contributes to meeting physical activity recommendations by fostering strength, flexibility, stress reduction, and psychosocial wellbeing (Stenner et al., 2016; Sorbie G. et al., 2020; Robinson et al., 2021a). Because of these positive aspects, exercise should be encouraged during the pandemic as long as keeping a social distance is possible. Thus, we propose a rethink of golf course closures in times of a pandemic to keep the middle-aged to older strata of the population active (Stenner et al., 2016). Research suggests that golf is an outdoor sport, where crowding is avoidable, social distancing feasible, and the infection risk negligible if effective hygiene concepts are in place, as already shown in a recently published study on the infection risk in a professional golf event (Robinson et al., 2021b). From a public health perspective, policymakers and decision-makers should promote participation in golf and avoid golf course closures.

## Limitations

The present study's findings should be contextualized in view of some methodological limitations.

First, in view of constantly changing national and regional restrictions, we compared the perceptions of golfers in the time before the start of the COVID-19 crisis in March 2020 to the time after. Therefore, this study did not focus on distinct periods of closing and re-opening of golf courses in the different German-speaking countries, mainly Austria and Germany.

Second, we relied on self-reports, which introduced recall bias and potentially lead to overestimating or underestimating the frequency of physical activity. It is already known from several other studies that all levels of physical activity from low to high were lower due to COVID-19 curfews, while sedentary behavior increased (Ammar et al., 2020a; Gallo et al., 2020; Pieh et al., 2020; Eek et al., 2021; Haider et al., 2021; Katewongsa et al., 2021). Thus, this assessment mainly focused on the shift of modes of activity from indoor to outdoor environments in golfers, not the activity level itself. In our study, we did not ask for the intensity of physical activity, presence of disability of problems with walking, or the use of electronic golf charts. These data could be of relevance for adjusting alternative modes for an active life in times of restricted access to outdoor and indoor sports facilities.

Third, our study sample consisted of a higher proportion of men and the average age of 55 years of our participants was similar to age patterns published in other studies among golfers (Luscombe et al., 2017; Murray et al., 2017; Sorbie G. G. et al., 2020; Sorbie et al., 2021; Robinson et al., 2021a,b). Although we did not study a representative sample of the general population, the sociodemographic characteristics of our study sample very likely mirror the typical golfer in the surveyed German-speaking golf community when compared with those of related studies.

Fourth, internet access was necessary to participate in the survey, potentially introducing a selection bias. The anonymous nature of online surveys did not allow for examining potential motives for nonresponse. Such investigation mode and sampling method potentially introduced sampling and selection bias, thus reducing the representativeness and generalizability of our findings. Future studies should employ randomized sampling approaches, and administrate both online and offline surveys to address all socioeconomic strata of the golf community. Further research is needed to cover these aspects in detail, also using advanced statistical procedures to uncover so far unclear effects of sociodemographic variables of golfers on their health and wellbeing. To elucidate the reasons for the changed life satisfaction of golfers during the COVID-19 pandemic, future research should use in-depth interviews, mixed-method assessments, and longitudinal study designs.

## Conclusions

Golf is a sport predominantly played outdoors in large areas. Thus, given the positive impact of golf on health and wellbeing mediated by low to moderate regular physical activity in natural environments, policymakers might consider avoiding golf course closures as far as is epidemiologically reasonable. Increasing and maintaining fitness in the general population, but especially in the elderly population and in light of aging populations, are of utmost importance in this pandemic, but also in any future one.

## Data Availability Statement

The dataset presented in this study can be found in the online repository Zenodo: https://zenodo.org/record/6406122.

## Ethics Statement

The studies involving human participants were reviewed and approved by Medical University of Vienna, Austria. The participants provided their written informed consent to participate in this study.

## Author Contributions

DH: conceptualization, project administration, and validation. DJ, MA, and DH: data curation, investigation, resources, software, writing—original draft, and writing—review and editing. DJ and DH: formal analysis. MA and DH: methodology and supervision. All authors have read and agreed to the published version of the manuscript.

## Conflict of Interest

The authors declare that the research was conducted in the absence of any commercial or financial relationships that could be construed as a potential conflict of interest.

## Publisher's Note

All claims expressed in this article are solely those of the authors and do not necessarily represent those of their affiliated organizations, or those of the publisher, the editors and the reviewers. Any product that may be evaluated in this article, or claim that may be made by its manufacturer, is not guaranteed or endorsed by the publisher.

## Footnotes

^1^SoSci Survey. Available online at: https://www.soscisurvey.de/ (accessed December 17, 2021).

^2^Perfect Eagle Golf. Available online at: https://www.perfect-eagle.com/community/golf-im-lockdown-umfrage/ (accessed December 17, 2021).

^3^Austrian Golf Association. Available online at: https://www.golf.at/magazin/sport-news/national/20210414-meduni-wien-umfrage-golf/ (accessed December 17, 2021).

^4^German Golf Association. Available online at: https://www.golf.de/publish/panorama/panorama-news/60228621/fuenf-minuten-fuer-die-wissenschaft (accessed December 17, 2021).

^5^Eurobarometer Sport and Physical Activity. Available online at: http://eose.org/wp-content/uploads/2014/03/european-barometer-survey_334_en_20101.pdf (accessed December 17, 2021).
